# Case report: Widely split P' waves in a patient with focal atrial tachycardia

**DOI:** 10.3389/fcvm.2023.1303200

**Published:** 2024-01-11

**Authors:** Hao Jiang, Zhongbao Ruan, Yin Ren, Xiangwei Ding

**Affiliations:** Department of Cardiology, The Affiliated Taizhou People’s Hospital of Nanjing Medical University, Taizhou School of Clinical Medicine, Nanjing Medical University, Taizhou, China

**Keywords:** focal atrial tachycardia, electrocardiography, electrophysiology, left atrial appendage, P' waves

## Abstract

**Background:**

Widely split P waves in sinus rhythm have been reported previously. However, widely split P' waves in focal atrial tachycardia (AT) on a surface electrocardiogram (ECG) have rarely been reported. The electrophysiological mechanism is relatively difficult to clarify, requiring a electrophysiological study.

**Case summary:**

A 67-year-old patient, who had undergone two radiofrequency ablations for atrial fibrillation, presented with recurrent palpitation. During the palpitation episode, the 12-lead ECG showed AT with a 3:1 atrioventricular conduction rate. P' waves were markedly prolonged in duration and widely split in morphology. An electrophysiological study showed that the tachycardia arose from the left atrial appendage (LAA) and was conducted through two distinct pathways. The impulse of one pathway was transmitted solely via the superior part of the atrium, including the Bachmann bundle. The second pathway was conducted via the coronary sinus and transmitted the impulse from the LAA to the ventricle. After the site showed that the earliest activation was ablated, repeated intravenous infusion of isoprenaline and programmed atrial stimulation did not induce tachycardia.

**Conclusion:**

Widely split P' waves in AT indicate intra- and interatrial conduction blocks, which can be easily overlooked due to the presence of low-voltage areas. Therefore, an electrophysiological study is crucial for identifying the origin of the tachycardia and elucidating the mechanistic details.

## 1 Introduction

The P wave represents depolarization propagation in the left and right atria. Electrophysiological abnormalities, including disturbed activation and conduction, illustrate various P wave morphologies, among which a prolonged P wave is common (1). Rarely, an extremely prolonged P wave can take on the morphology of widely split P waves, indicating delayed conduction between the two atria, including the Bachmann bundle zone. Previously, widely split P waves have been reported in sinus rhythm (2, 3). Nevertheless, no report about the splitting of P' waves in atrial arrhythmia has been published to date.

In this study, we report a case of wide splitting of P' waves in a patient with focal atrial tachycardia (AT) and clarify the details of the electrical mechanism.

## 2 Case presentation

**Figure 1:**
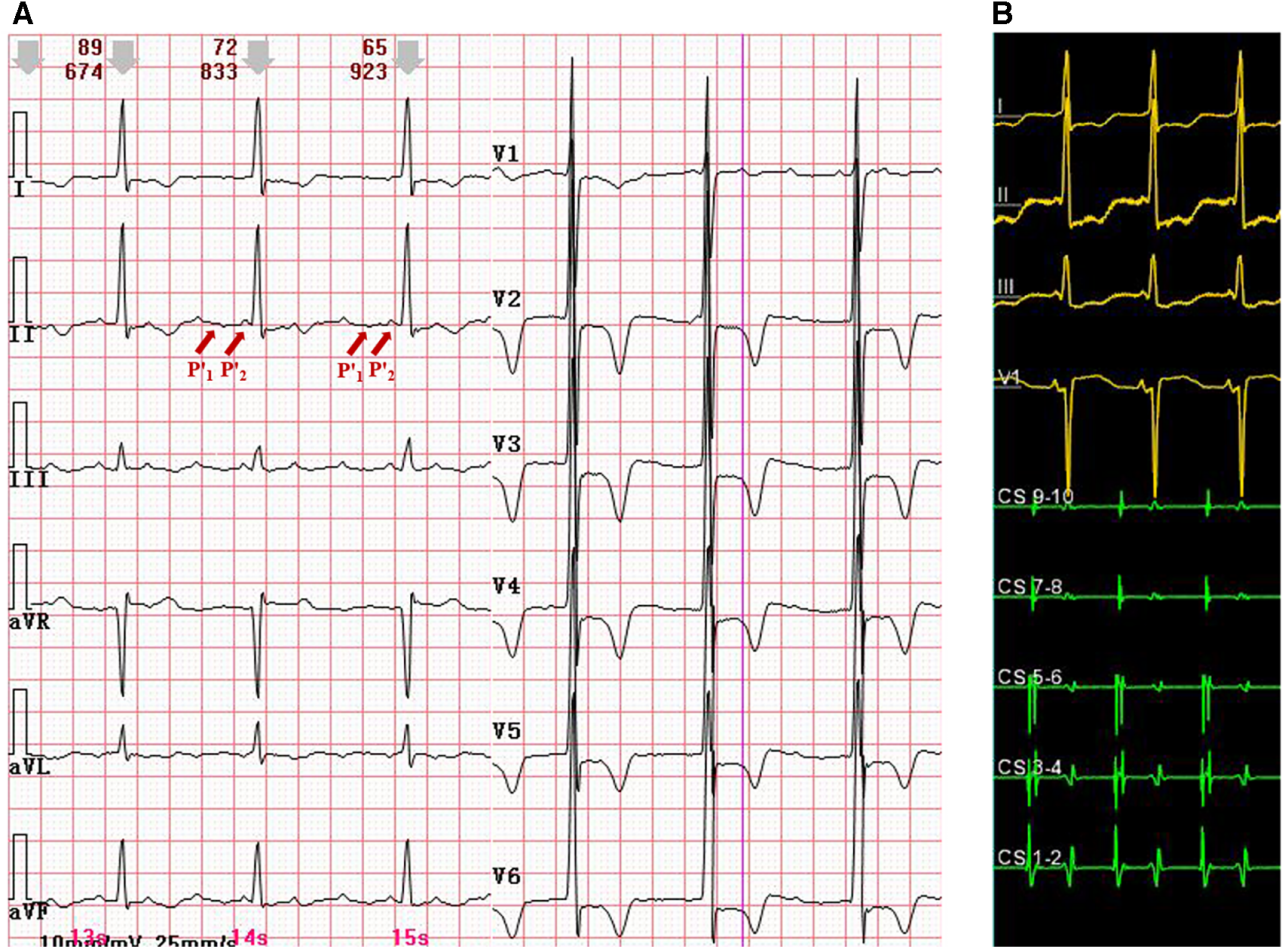
(**A**) A 12-lead ECG with atrial tachycardia and widely split P' waves. P'_1_ morphology was biphasic, and P'_2_ morphology was positive in the inferior leads. The P'_2_ wave almost merged with the QRS complex. (**B**) An intracardiac ECG during atrial tachycardia. The earliest atrial activation was at CS1-2. CS, coronary sinus; ECG, electrocardiogram.

A 67-year-old woman presented with recurrent palpitation, which had initially emerged 2 years earlier. The patient had previously undergone two radiofrequency ablations for atrial fibrillation. Other than pulmonary vein isolation, line ablations were performed in the anterior wall of the left atrium, mitral isthmus, and cavotricuspid isthmus. A transthoracic echocardiogram demonstrated no enlargement of the atria and a left ventricular ejection fraction of 77%. The 12-lead electrocardiogram (ECG) recorded during a palpitation episode showed AT with a 3:1 atrioventricular conduction rate. P' waves were prolonged in duration (ca. 220 ms) and were widely split in morphology (P'_1_ wave morphology was biphasic and P'_2_ wave morphology was positive in the inferior leads; the P'_2_ wave almost merged with the QRS complex) (Figure 1).

**Figure 2:**
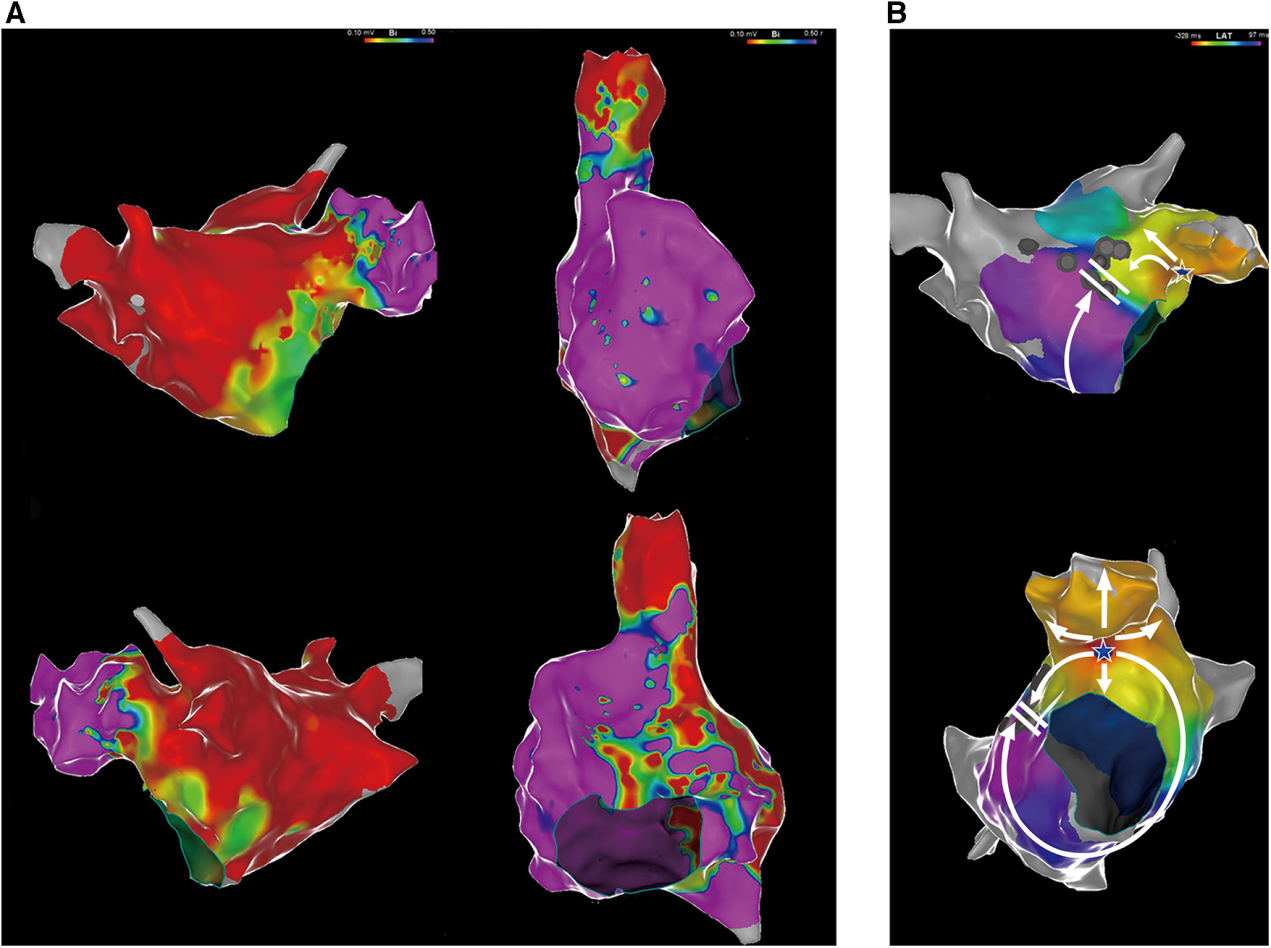
(**A**) Voltage mapping of the left and right atria. A low-voltage area was defined as contiguous areas of bipolar voltage <0.5 mV. Purple represents areas with relatively normal voltages, and the other colors represent low-voltage areas. (**B**) An electrophysiological mapping of the left atrium with atrial tachycardia. The white star represents the earliest activation point in the LAA. The white arrows represent the direction of atrial activation. LAA, left atrial appendage.

Then, an electrophysiological study was performed. The intracardiac ECG showed that the earliest atrial activation developed in the distal coronary sinus (CS), indicating that focal AT originated from the left atrium (Figure 1). Voltage mapping demonstrated a wide distribution of low-voltage areas in the left atrium, suggesting slow conduction between the left and the right atria, including the Bachmann bundle zone (Figure 2). The propagation map during atrial tachycardia (Supplementary Video S1) indicated that focal AT arose from the left atrial appendage (LAA). The left atrial anterior line was blocked (Figure 2).

**Figure 3:**
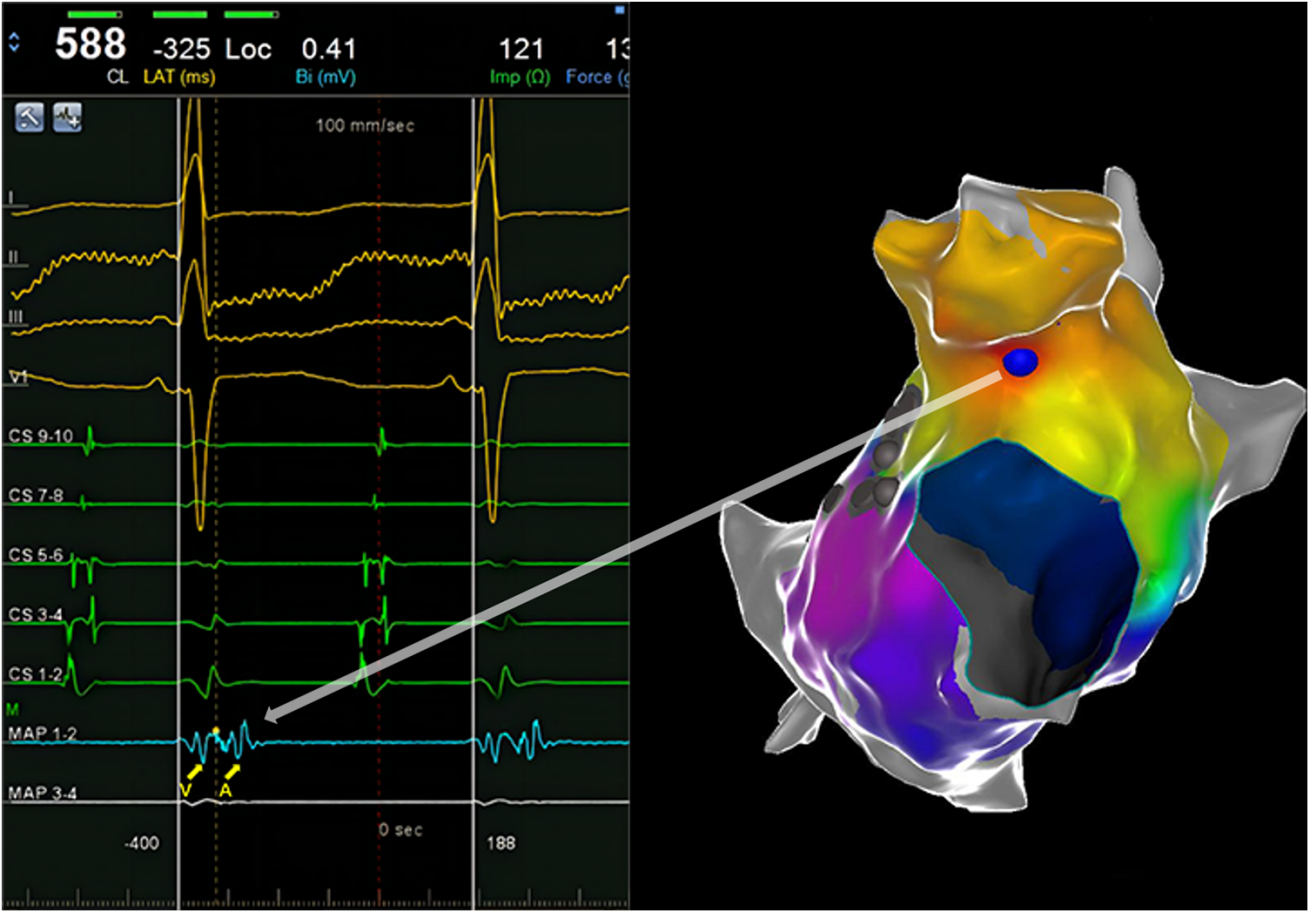
An intracardiac ECG shows the earliest activation. The yellow dotted line and blue ball represent the earliest activation.

The tachycardia originated from the LAA, as confirmed by the mapping (Figure 3). Ablation was applied at the site of earliest activation, with power limited to 30 W and a temperature below 55°C. Following the gradual prolongation of the tachycardia cycle length, atrial tachycardia was terminated. Repeated intravenous infusion of isoprenaline and programmed atrial stimulation did not induce AT.

After ablation, the palpitation episode did not recur. The ECG recorded after the procedure showed a P wave in sinus rhythm, while the widely split P waves appeared to be absent (Supplementary Figure S1). Considering the presence of intra- and interatrial delayed conduction, it was possible that the left atrial P wave may have merged with the QRS complex.

## 3 Discussion

**Figure 4:**
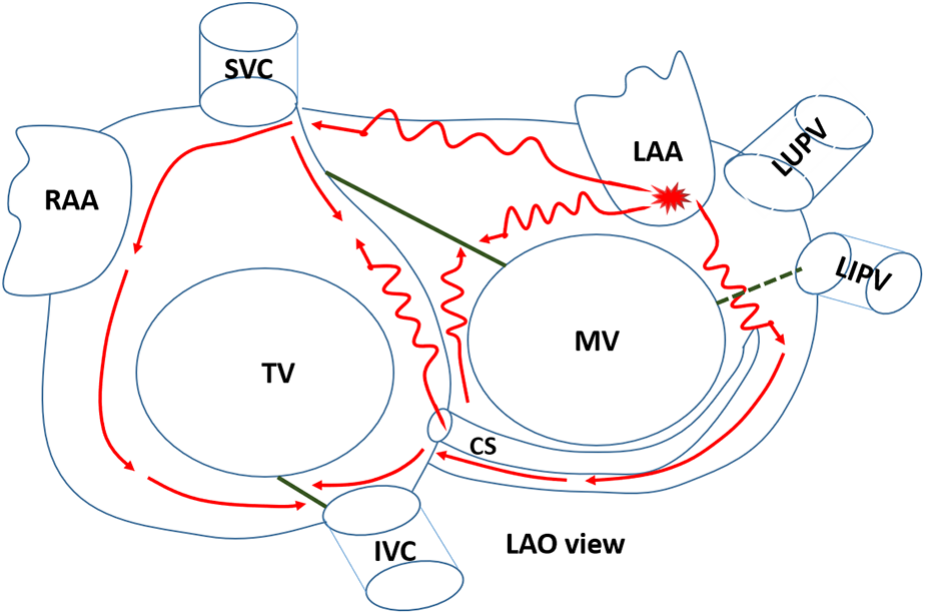
The detailed mechanism underlying the tachycardia. The focal atrial tachycardia originated from the LAA and was conducted through two distinct pathways. LAO, left anterior oblique; CS, coronary sinus; LAA, left atrial appendage; RAA, right atrial appendage; MV, mitral valve; TV, tricuspid valve; SVC, superior vena cava; IVC, inferior vena cava; LUPV, left superior pulmonary vein; LIPV, left inferior pulmonary vein. The green solid lines represent the conduction block line. The green dashed line represents the failure of conduction in the completely blocked line.

In this study, we illustrated the detailed mechanism underlying widely split P' waves in a patient with focal AT (Figure 4). The tachycardia arose from the LAA and was conducted through two distinct pathways. The impulse of one pathway was transmitted solely via the superior part of the atrium, including the Bachmann bundle, owing to a block in the anterior line. The conduction appeared to be relatively slow, which was associated with wide low-voltage areas. The second pathway was conducted via the CS, transmitting the impulse from the LAA to the ventricle. Consequently, the CS1-2 electrodes were activated earlier than the CS9-10 electrodes. Due to previous ablation in the mitral isthmus (not completely blocked), the impulse from the LAA was conducted very slowly through the mitral isthmus to CS1-2. Notably, activation through the CS was blocked in the cavotricuspid isthmus and was disrupted by the low-voltage area in the interatrial septum, leading to the right atrium mainly activated by the impulse via the Bachmann bundle (although the conduction of the Bachmann bundle was very slow), resulting in a positive P'_2_ wave in the inferior leads. The aforementioned observations may explain the widely split P' waves in the surface ECG.

In lead II of the surface ECG, the P'_1_ wave was biphasic with a dominant negative component. The amplitude of the positive component was reasonably low, likely due to a wide range of atrial scars in the anterior, roof, and posterior walls. The negative component of the P'_1_ wave was attributed to activation of the left atrial bottom wall and the right atrial septum. The activation of CS5-6 might indicate the electrical activity of the left atrial bottom, and CS9-10 might indicate the onset of activation of the right atrial septum. As shown in Supplementary Figure S2, the time interval from CS5-6 to the onset of the P'_2_ wave in the surface ECG was 125 ms, and the time interval from CS9-10 to the onset of the P'_2_ wave in the surface ECG was 109 ms. The activation of CS5-6 and C9-10 occurred within the duration of the negative component of the P'_1_ wave in the surface ECG. Therefore, activating the left atrial bottom wall and the right atrial septum contributes to P'_1_ wave formation.

Focal AT is a relatively rare arrhythmia, accounting for approximately 10% of all supraventricular tachycardias (4). However, the LAA is an uncommon site of AT origin, accounting for 2.1% of all focal AT cases (5). Identifying its mechanism by surface ECG is difficult. Hence, an electrophysiological study is important to elucidate its origin. The electrophysiological features of focal AT arising from the LAA have been described, including an upright or biphasic P' wave in lead V1 and a negative P' wave in lead aVL (6). However, these typical P' wave morphologies were not observed in the case of our patient. This discrepancy might be attributed to aberrant atrial tachycardia conduction, possibly due to biatrial block. In addition, the P'_1_ wave was mainly negative in the inferior leads, suggesting a reduced presence of low-voltage zones in the bottom part as compared to the anterior part of the left atrium.

The P wave's morphology, amplitude, and duration reflect atrial activities. Analyzing the P wave in the surface 12-lead ECG provides insights into the anatomical structure and electrophysiological activity of the atrium. For example, the voltage of lead V1 is indicative of atrial activity. A higher voltage in lead V1 is associated with a more organized atrium, indicating a higher degree of atrial activation. Atrial arrhythmias with V1 voltages below 0.11 mV are correlated with a broader range of atrial scars, highlighting the anisotropy of conduction and notable atrial remodeling (7). In the case of our patient, the amplitude of the P'_1_ wave in lead V1 was fairly low during the surface ECG, indicating atrial remodeling and conduction block. This prediction was validated through voltage mapping, which revealed a wide range of low-voltage areas.

Recently, emerging digital tools for novel P wave analysis in surface ECGs, such as the tempospatial localization of atrial activity, have provided accurate insights into atrial activity. Locati et al. introduced CineECG, a tool that describes the anatomical and electrical structure of atrial activity using data from electrocardiograms and vectorcardiograms. CineECG is a valuable tool for quantifying the tempospatial localization of atrial activity. It vividly displays the formation and propagation of atrial activity digitally, relating the electrical pathway to cardiac anatomy (8). However, only normal atrial P waves were analyzed in that study. In our patient, the AT originated from a rare site and showed an unusually complex wavefront, including a highly interatrial conduction block, as validated by intracardiac ECG. A further study of novel P wave analyses in a rarely complex surface ECG is needed to gain more insights into atrial activity.

The wide splitting of P' waves could potentially be iatrogenic. Previously, line ablations had been performed in the anterior wall of the left atrium, the mitral isthmus, and cavotricuspid isthmus, altering the atrial conduction, which resulted in widely split P' waves. Previously, cases of proven iatrogenic widely split P' waves have been reported (9, 10). In our patient, split P' waves arose from a rare site of AT and the P'_1_ wave was easily overlooked due to the presence of low-voltage areas in the left atrium.

Our study had several limitations. First, no propagation mapping was done in the right atrium. An activation map of the right atrium during tachycardia episodes could provide a clearer understanding of its underlying mechanism. Regrettably, propagation mapping of the right atrium was not performed. We noticed this omission immediately following tachycardia termination. Therefore, we used an intravenous infusion of isoprenaline and programmed atrial stimulation. Unfortunately, the tachycardia could not be induced. We then conducted a detailed voltage mapping of the right atrium. The results revealed a scar zone in the cavotricuspid isthmus, due to previous ablation. A wide range of low-voltage areas was found in the right atrial septum. The roof and lateral walls appeared relatively healthy (Figure 2A). The P'_2_ wave was also positive in the inferior leads. Therefore, we speculated on the formation mechanism of the P'_2_ wave (see Figure 4). Based on the voltage mapping of the right atrium, the conduction time in the right atrial lateral wall was expected to be short due to normal voltage. In contrast, the conduction velocity in the septum of the right atrium should be slow due to the extensive distribution of the low-voltage zone. Unfortunately, the lack of a detailed right atrial propagation mapping made it challenging to accurately measure the activation time of the septal and lateral walls in the right atrium. However, the time interval from CS9-10 to the onset of the P'_2_ wave in the surface ECG was measured during tachycardia episodes (ca. 109 ms), as shown in Supplementary Figure S2. The activation of CS9-10 might represent the onset time of the right atrial septum, whereas the P'_2_ wave represents the activation of the roof and lateral walls of the right atrium. This time interval was relatively long, potentially reflecting just a part of the right atrial septum conduction time. The duration of the P'_2_ wave was merely 80 ms, partly showing the high velocity of conduction in the roof and lateral right atrium. Second, we did not perform entrainment pacing. Our patient had previously undergone anterior line ablation. Based on left atrial propagation mapping, perimitral flutter using a Bachmann bundle epicardial bypass could not be excluded entirely. Since “interface” connections are usually broad, a poor termination rate is observed with a progressive shift of the endocardial emergence as the patchy lesion expands (11). In our patient, centrifugal propagation was identified, and subsequent ablation just at the site of earliest activation could terminate the tachycardia. Therefore, the underlying mechanism of centrifugal propagation was most likely focal AT in our patient. However, entrainment pacing at an area opposite the breakthrough site indeed helps rule out macro-reentrant AT. Therefore, entrainment pacing will be performed when we encounter similar cases in the future.

## 4 Conclusion

Widely split P' waves in AT indicate intra- and interatrial conduction blocks, which are easily overlooked due to low-voltage areas. This report illustrates that an electrophysiological study is crucial for finding the origin of the tachycardia and identifying the details of the underlying mechanism.

## Data Availability

The raw data supporting the conclusions of this article will be made available by the authors, without undue reservation.
