# Supplementary material for: Case report: Widely split P' waves in a patient with focal atrial tachycardia
Source: Front Cardiovasc Med. 2024 Jan 11;10:1303200. doi: 10.3389/fcvm.2023.1303200 (PMC10808459; doi:10.3389/fcvm.2023.1303200)
Supplement: Supplementary file 1 [file Datasheet1.docx]

Case Report: Widely Split P' Waves in a Patient with Focal Atrial Tachycardia

Hao Jiang^1^, Zhongbao Ruan^1^, Yin Ren^1^, Xiangwei Ding^1^

^1^Department of Cardiology, The Affiliated Taizhou People’s Hospital of Nanjing Medical University, Taizhou School of Clinical Medicine, Nanjing Medical University, Taizhou 225300, China

*** Correspondence:**Xiangwei Ding, M.D.
dingxw1208@126.com

**Keywords: Focal atrial tachycardia, electrocardiography, electrophysiology, left atrial appendage, P' waves**

**Supplementary Appendix**

This appendix has been provided by the authors to give readers additional information about their work.

**List of Contents**

Page 1: Cover page

Page 2: List of contents

Page 3: Supplemental Figure 1

Page 4: Supplemental Figure 2

**
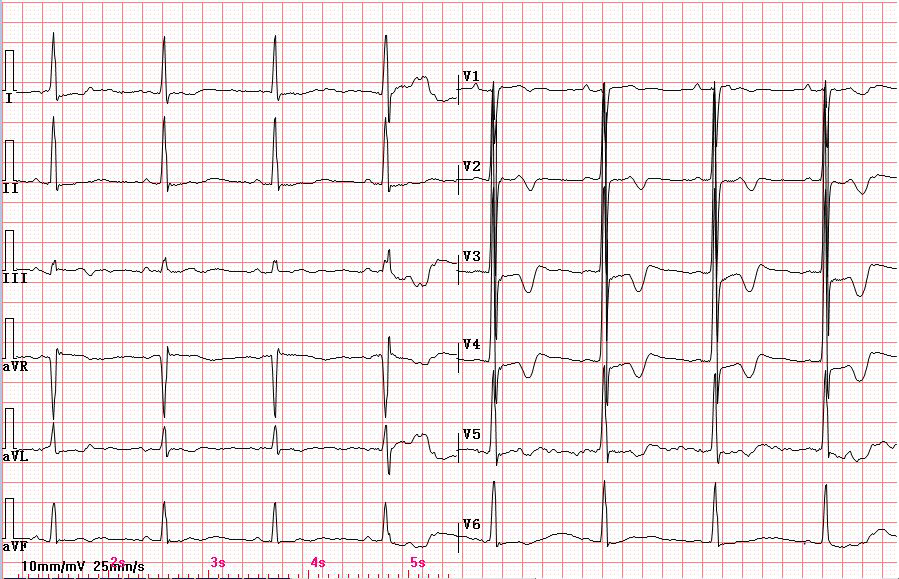
**

**Supplemental Figure 1.** Twelve-lead ECG with sinus rhythm.

**
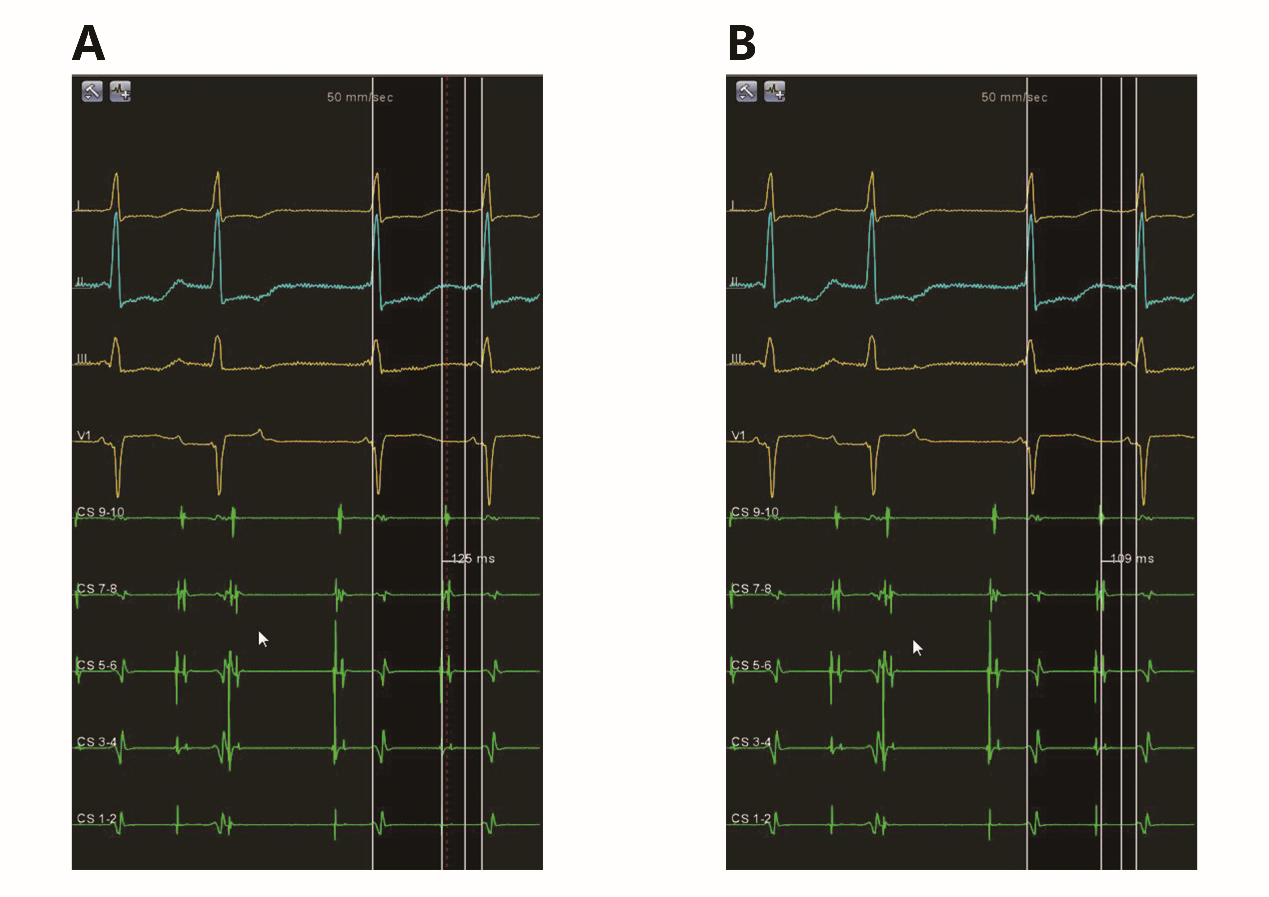
**

**Supplemental Figure 2.** Intracardiac ECG during atrial tachycardia. A: The time interval from CS5-6 to the onset of P'_2_ wave in surface ECG was 125ms. B: The time interval from CS9-10 to the onset of P'_2_ wave in surface ECG was 109ms.
